# Metofluthrin: investigations into the use of a volatile spatial pyrethroid in a global spread of dengue, chikungunya and Zika viruses

**DOI:** 10.1186/s13071-017-2219-0

**Published:** 2017-05-30

**Authors:** Tamara S. Buhagiar, Gregor J. Devine, Scott A. Ritchie

**Affiliations:** 1 College of Public Health, Medical and Veterinary Sciences, James Cook University, PO Box 6811, QLD, Cairns, 4870 Australia; 2 Mosquito Control Laboratory, QIMR-Berghofer Medical Research Institute, QLD, Brisbane, 4006 Australia

**Keywords:** *Aedes aegypti*, Spatial repellents, Metofluthrin, Pyrethroids, Mosquito control, Dengue fever, Zika virus, Chikungunya

## Abstract

**Background:**

Metofluthrin reduces biting activity in *Aedes aegypti* through the confusion, knockdown, and subsequent kill of a mosquito. A geographical spread in dengue, chikungunya, and Zika viruses, increases intervention demands. Response to a Zika outbreak may require a different strategy than dengue, as high-risk individuals, specifically pregnant women, need to be targeted.

**Methods:**

In semi-field conditions within a residential property in Cairns, Queensland, the impacts of metofluthrin on biting behaviour of free-flying Wolbachia-infected *Ae. aegypti* were evaluated.

**Results:**

Mortality in *Ae. aegypti* exposed to metofluthrin over a 22 h period was 100% compared to 2.7% in an untreated room. No biting activity was observed in mosquitoes up to 5 m from the emanator after 10 min of metofluthrin exposure. Use of metofluthrin reduced biting activity up to 8 m, regardless of the host’s proximity (*near* or *far*) to a dark harbourage area (HA) (*P* < 0.0001 and *P* = 0.006), respectively. In the presence or absence of the metofluthrin emanator, the host was most likely bitten when located immediately next to a HA (within 1 m) *versus* 8 m away from the HA (*P* = 0.006). The addition of a ceiling fan (0.8 m/s airflow) prevented all biting activity after 10 min of metofluthrin exposure. Previously unexposed *Ae. aegypti* were less likely to reach the host in a metofluthrin-treated room $\Big(\overset{-}{X}$= 31%) compared to an untreated room ($\overset{-}{X}=100\%$) (*P* < 0.0001). In a treated room, if the mosquito had not reached the host within 30 s, they never would. Upon activation, the time required for metofluthrin to infiltrate *protected* locations within a room causing knockdown in caged mosquitoes, required more time than *exposed* locations (*P* < 0.003); however *exposed* and *protected* locations do eventually reach equilibrium, affecting mosquitoes equally throughout the room.

**Conclusion:**

Metofluthrin is effective in interrupting indoor host-seeking in *Ae. aegypti.* Metofluthrin’s efficacy is increased by centrally locating the emanator in the room, and by using a fan to increase airflow. Newly treated rooms may require a period of 2–4 h for sufficient distribution of the metofluthrin into *protected* locations where mosquitoes may be resting.

## Background

*Aedes aegypti* (L.) has a ubiquitous distribution throughout urban areas in tropical regions of the world and is the primary vector of dengue (DENV), yellow fever (YFV), chikungunya (CHKV), and Zika (ZIKV) viruses [1–3]. In the last 50 years, the public health impact of DENV and CHKV has increased dramatically, with both diseases spreading geographically and increasing in incidence [4]. Recently, ZIKV has had major impacts on public health, particularly in South America, with increasing incidence of imported and locally acquired cases occurring globally [5, 6]. Countries with confirmed mosquito transmission of ZIKV have so far been limited to South and Central America, the Pacific Islands, including Papua New Guinea, and most recently in the southern United States, in Miami Florida [7].

In Australia, *Ae. aegypti*’s current distribution is limited to Queensland [8] and is most abundant in the far north where outbreaks of DENV occur regularly as a result of imported cases [9–11]. Since 2011, 1842 cases of DENV, and 49 cases of CHKV have been reported, and 37 cases of ZIKV since 2015 [12]. Local transmission of both CHKV and ZIKV have yet to be observed, however regular outbreaks of DENV, illustrate the potential for outbreaks of both of these viruses.

Studies have shown reduced vector competence in *Ae. aegypti* infected with the *w*Mel strain of *Wolbachia* for DENV, CHKV, and YFV [13, 14]. More recent evidence also shows reduced vector competence in a Colombian strain of *w*Mel *Ae. aegypti* using ZIKV-infected mice [15]. In parts of far north Queensland, a project involving the release of *w*Mel*-*infected *Ae. aegypti* is underway, whereby *w*Mel-infected male and female *Ae. aegypti* are released in large numbers to replace the wild population [16]. If successful, local transmission of DENV in Australia, could be significantly reduced. However, it should be noted that high densities of *Ae. aegypti* with poor vector competence, have successfully sustained arbovirus outbreaks in the past, as seen with YFV [17].

Indoor residual spraying (IRS) remains the current best practice in limiting DENV outbreaks [9]. It is labour-intensive, and in Queensland, is usually limited to viraemic contact addresses, their nearest neighbours, and other identified high-risk properties (i.e. hostels) [9, 18]. It is also limited by whether or not the house owner grants permission to enter the premises [18], and in an outbreak, labour resources to apply IRS can become rapidly exhausted, leaving large parts of the population vulnerable for periods of time. Currently, Queensland homeowners are encouraged to use commercially available repellents and indoor surface sprays both during and outside an outbreak, to control DENV vectors [19]. However, acceptance, compliance, and efficient use of this method will vary between individuals. Targeted indoor residual spraying for DENV control works on the principal that adult *Ae. aegypti* are endophilic and are highly attracted to dark objects and shady areas. Treatment of these areas with residual insecticides can kill adult mosquitoes seeking to rest on treated surfaces, interrupting transmission [9, 20].

There is a gap in this method that needs to be addressed, and that is, “how do we protect an uninfected person from being bitten by an infected mosquito that newly enters a dwelling?” This question becomes particularly important with the global emergence of ZIKV and protecting pregnant women from infective bites. The response during a ZIKV outbreak may, in fact, look quite different from the strategies that would be applied to DENV. In addition to high-risk properties, high-risk individuals (e.g. pregnant women) will also need to be targeted, making interventions such as widespread IRS difficult to employ. This is where alternative strategies that are highly effective, safe, commercially available, inexpensive, easy to use, and receive wide public acceptance, become very important.

Commercially available spatial repellents, such as mosquito coils and vaporizer mats are well documented in repelling mosquitoes from human biting [21, 22]. Unfortunately, both of these products are limited by their degree of efficacy, the requirement for a heat source to vaporize their active ingredient [22, 23], and their limited use indoors [24]. Studies have shown varying susceptibility to pyrethroid-based mosquito coils between different mosquito species, and within a species, specifically *Ae. aegypti* [22, 23]. A study by Liu et al. [25] found a large suite of volatile organic compounds, including carcinogens and suspected carcinogens in the mosquito coil smoke. In fact, mosquito coil smoke produced the same amount of fine and ultrafine particulate matter mass as burning 75–137 cigarettes [25]. Recently, a study in Ghana found mosquito coils had little effect on reducing malaria incidence. They did, however, present a potential respiratory risk factor that requires further investigation [26]. The need for safer, more effective, inexpensive, and simple, spatial repellents remains.

Investigations into the insecticidal activity of nonchrysanthemic acid esters with high vapour activity at ambient temperature [27] have resulted in the development of a synthetic pyrethroid commonly known as metofluthrin. Metofluthrin is a volatile pyrethroid insecticide that has been shown to be extremely effective in reducing biting in *Ae. aegypti* [28–30]. The efficacy of this compound lies in its safe and effective use indoors; it accumulates within a room rapidly and affects biting activity within a relatively short period of time [31]. Often referred to as a “spatial repellent,” its mode of action is not through repellency at all, but rather through the disruption in orientation towards the host (preventing biting), knockdown, and kill of *Ae. aegypti* [21, 31].

In this study, we investigated the use of a 10% active ingredient (AI) metofluthrin emanator (Sumitomo Chemical Australia Pty Ltd) indoors by determining: its effective range and impacts on mosquito biting and mortality; its efficacy against new mosquitoes entering a treated room; and the potential for protection of mosquitoes resting in cryptic harbouring areas within a treated room. Based on metofluthrin studies to date*,* this product may provide a complimentary solution to fill the current gaps in handling arbovirus outbreaks transmitted by *Ae. aegypti*, by interrupting disease transmission to the most vulnerable.

## Methods

### Experimental setting

**Fig. 1:**
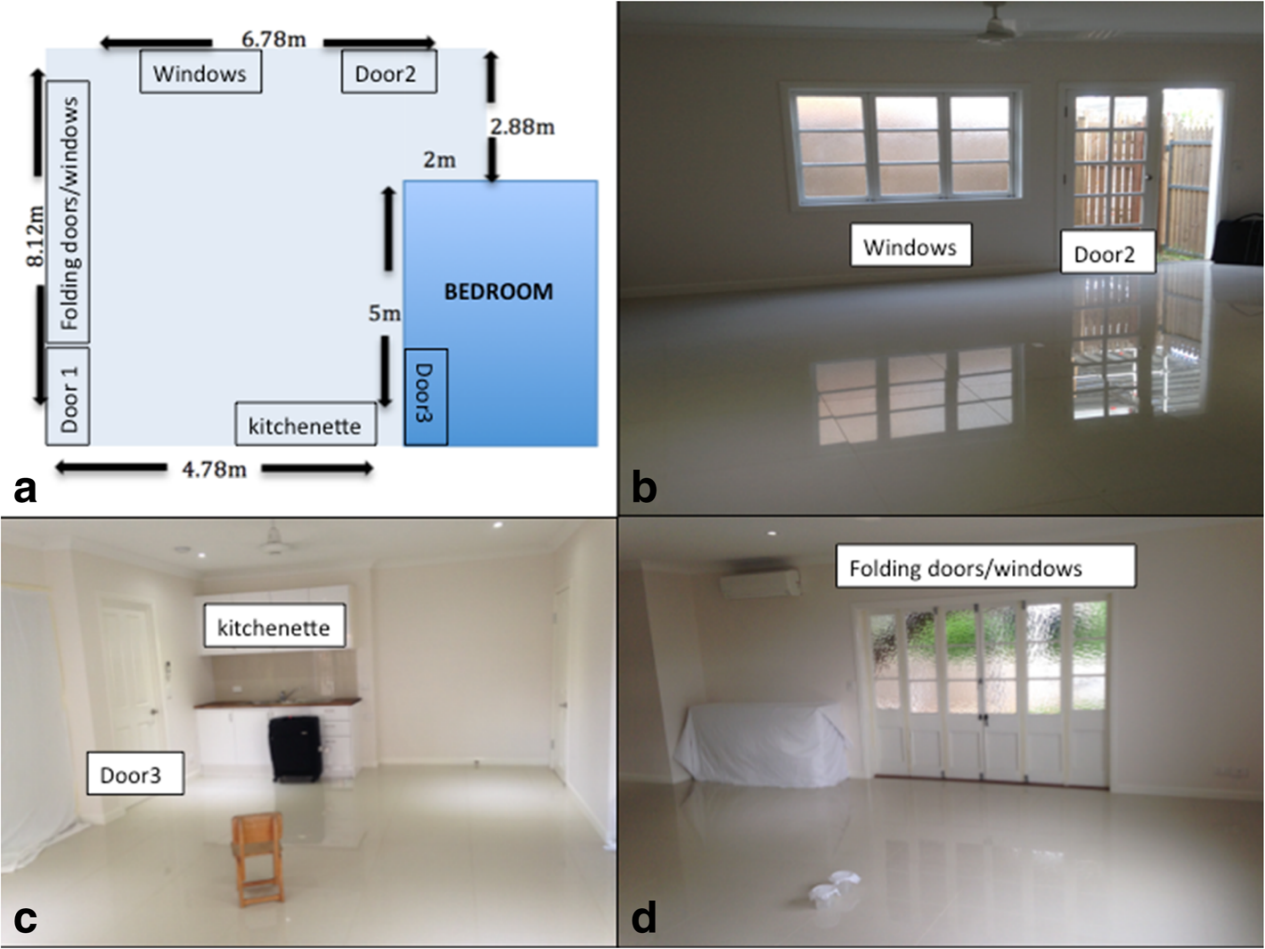
**a** Floor plan of the large experimental room (111 m^3^). **b-d** 360-degree view of the large experimental room

All experiments took place within a large enclosed living area (111 m^3^) below a typical Queenslander-style house in Cairns, Queensland, Australia (Fig. 1a–d). Throughout this study we will use the term “harbourage area” (HA) to describe an object, usually a suitcase, that is black or dark surfaced. Since *Ae. aegypti* have a tendency to gather and rest near, and on dark surfaces, we use these HAs to visually bait or direct the mosquito to a chosen part or parts of the room throughout this study.

**Fig. 2:**
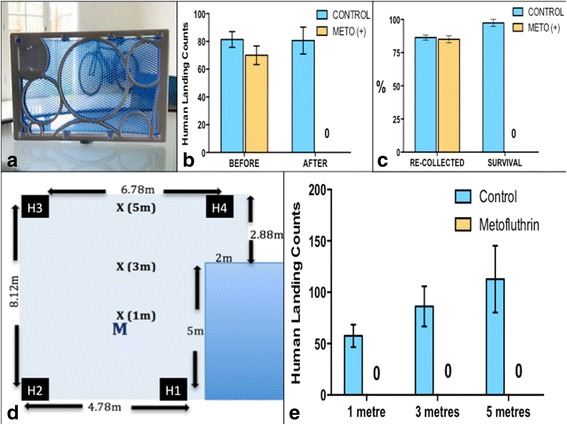
**a** 10% AI metofluthrin emanator (Sumitomo). **b** Mean (± SE) human landing count before and after control and treatment (METO+) groups. **c** The mean (± SE) number of mosquitoes recollected after 22 h, and the mean (± SE) survival (%) of recollected mosquitoes. **d** Experimental room set-up indicating locations of HLCs, the metofluthrin emanator, and the harbourage sites. **e** Mean (± SE) human landing counts at 1, 3 and 5 m proximity to the emanator

The metofluthrin product used in this study was a small (9.5 × 15 × 1 cm), plastic frame containing a polyethylene mesh which the 10% AI *w*/w formulation is incorporated into (Sumitomo Chemical Australia Pty Ltd., Sydney, Australia) [31] (Fig. 2a). In previous longevity studies of this emanator, its efficacy was sustained up to 20 days [31].

### Rearing, sexing, caging and blood-feeding mosquitoes

A Cairns colony (F1) of *Ae. aegypti* infected with *w*Mel *Wolbachia,* derived from field populations, was used throughout the experiments. The colony was reared in a controlled temperature room at 25 °C and maintained at 70% relative humidity. Wolbachia-infected mosquitoes were used to avoid accidental introduction of uninfected mosquitoes in an area where the Eliminate Dengue program had established *w*Mel in *Ae. aegypti.* Female mosquitoes were separated as pupae by size and then allowed to emerge into 500 ml containers or into a BugDorm (30 × 30 × 30 cm), depending on the requirements of the experiment. A 10% honey pad was provided and removed 24 h prior to use in experiments. During rearing, when blood meals were required, mosquitoes were offered a blood meal by resting the back of a human leg on top of the cage or container.

### Human landing counts

During experiments where a human landing count (HLC) was performed, the lower half of both legs were exposed, and only landing/biting attempts on the exposed lower legs were recorded.

### Twenty-two-hour exposure: recovery and survival (fan off)

To observe the impact of metofluthrin on *Ae. aegypti* survival, forty female *Ae. aegypti* were released into a large room (111 m^3^) (Fig. 1) for a 22-h period in both a metofluthrin-treated and an untreated room. An initial HLC was performed immediately prior to each treatment and replication to confirm the fitness of the released mosquitoes. During the treatment replications, the metofluthrin emanator was activated by removing the emanator from the packaging and placing it in centrally within the room to volatilize immediately following the pre-treatment HLC. After 22 h, all live mosquitoes were collected from the room using an insect net, and all knocked down mosquitoes were gently collected into paper towel-lined 500 ml cups using forceps. Observations for mortality and recovery occurred up to 3 h after the completion of each replication. A total of 5 replications were completed. Data from the experiment were analyzed using a Mann-Whitney test in Prism 6.

### Effective spatial range of metofluthrin (fan off)

In a large room (111 m^3^) with four HAs placed in each corner of the room, and a centrally located metofluthrin emanator (Fig. 2d), 10 female *Ae. aegypti* were released for each replicate, and biting activity was observed over a 5-min period each at 1, 3 and 5 m intervals from the emanator. Once the mosquitoes were released, they were allowed to settle over a 10-min period. For each replication, a control was performed prior to the treatment. For the treatment, the metofluthrin emanator was activated for 10 min prior to the first HLC. A total of 4 replications were completed. Between each replicate, the room was aired out using strong fans with all windows and doors open over a 2-h period. Mosquitoes were not removed or replaced when rotating through the three locations (1, 3 and 5 m) in the room, however, the starting point for each replication was rotated. Data were analyzed using a Mann-Whitney test in Prism 6.

### The effect of host and metofluthrin location relative to harbourage areas on human landing counts (fan on *vs* fan off)

#### Fan off

**Fig. 3:**
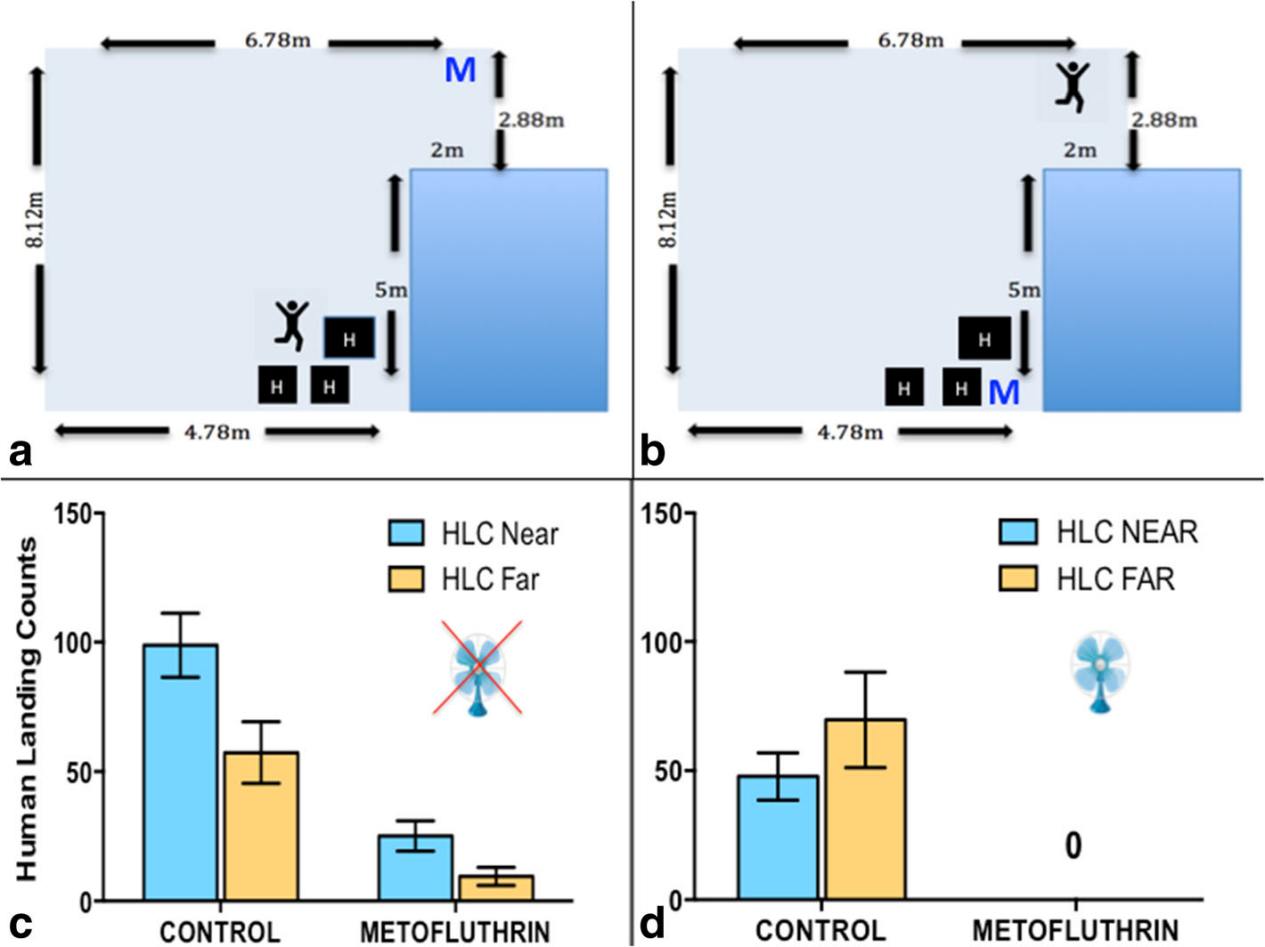
**a** Room set-up for metofluthrin “far” from the HA and host “near,” where “H” indicates clusters of harbourage items and “M” indicates the location of the metofluthrin. **b** Room set-up for metofluthrin “near” the HA and host “far.” **c** HLCs (± SE) with the fan off. **d** HLCs (± SE) with the fan on

Here we visually baited female *Ae. aegypti* with one large HA, at one end of the room (Fig. 3a, b). We looked at the influence of the proximity of the host and the metofluthrin emanator to HAs on HLCs. In order to do this, we performed two treatments: (i) when the host was *near*, this meant that the host was within 1 m of the HA, and the emanator was placed on the opposite end of the room, 8 m away from the HA and the host; (ii) when the host was *far* from the HA, the host would be 8 m from the HA on the opposite end of the room, and the emanator located immediately next to the HA. During the control replicates for each of these treatments, *near* and *far* refer to the position of the host relative to the HA without the presence of an active emanator in the room.

For each replication, 10 *Ae. aegypti* females were released into the room (111 m^3^) and allowed to settle for 10 min. A 5-min HLC was performed prior to each treatment (activation of the metofluthrin emanator in the room), as a control, corresponding to the host location (*near* or *far*) of the subsequent treatment. Once the control was completed, the mosquitoes were again allowed 5 min to settle prior to activating the metofluthrin emanator. The metofluthrin, once activated, was allowed to volatilize for 10 min prior to the HLC. A total of 14 replications were performed. Halfway through the replications, the HAs were moved to the opposite end of the room to eliminate a location effect. Data were analyzed in SPSS using a negative binomial regression analysis.

#### Fan on

In a related experiment, we repeated the above protocols with the addition of a ceiling fan. We wanted to see if a ceiling fan on its lowest setting (0.8 m/s airflow when standing directly below the fan, measured with a Kestrel anemometer, 1000 model) would aid in the circulation of the metofluthrin throughout the room, thus increasing its efficacy against host-seeking *Ae. aegypti*. A total of 4 replications were completed. Data were analyzed in Prism 6 using a Mann-Whitney test.

### Introduction of a new mosquito to a treated room (fan off)

**Fig. 4:**
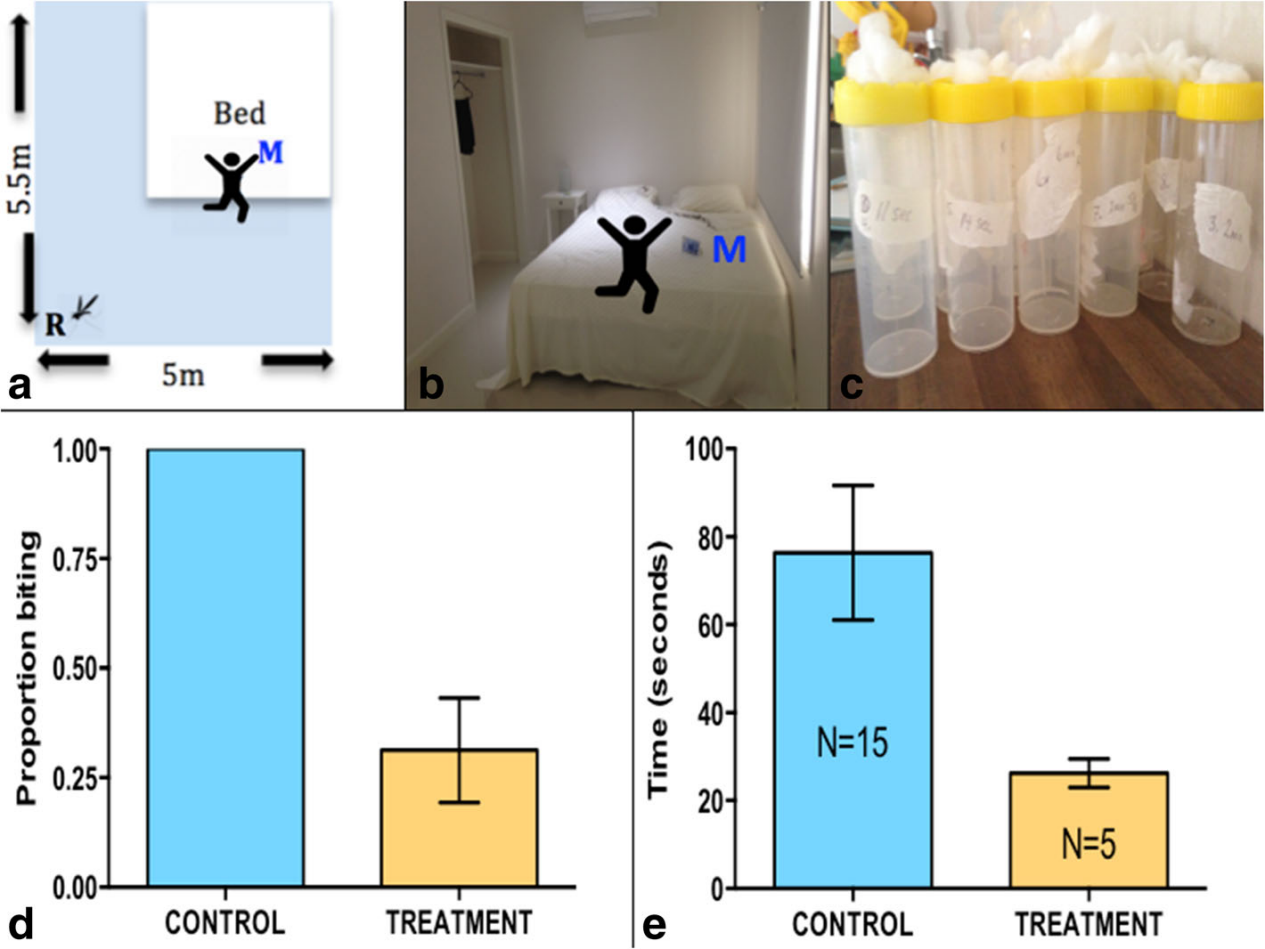
**a** Bedroom set-up (70 m^3^). “R” indicates the mosquito release point. Metofluthrin emanator indicated by “M,” next to the host. **b** Photo of bedroom set-up. **c** Containers individual mosquitoes were held in and released from. **d** Mean percent (± SE) of *Ae. aegypti* released that reached the host within 10 min. In the treatment group, 31.3% of all mosquitoes reached the host. **e** Of all mosquitoes that reached the host post-release, the mean (± SE) number of seconds required

In a 70 m^3^ bedroom, attached to the large main room (Fig. 4a, b), one female *Ae. aegypti* was released for each replication of the control (*n* = 16) and treatment (*n* = 16). For the treatment, a metofluthrin emanator was allowed to volatilize centrally within the room (without a fan) for a 30-min period (Fig. 4a, b). Each mosquito was released from a small covered (200 ml) cylindrical container at the corner of the room, approximately 3 m from the host (Fig. 4a, b). The host sat centrally in the room, at the end of the bed, next to the activated emanator (Fig. 4a, b). The time required for the mosquito to reach the host was recorded, for up to 10 min, for both the control and treatment. Whether or not the mosquito reached the host was also recorded. A total of 16 replications were completed. Data were analyzed in Prism 6 using a Fisher’s exact test.

### Impact of protected mosquito resting areas on efficacy (fan on)

**Fig. 5:**
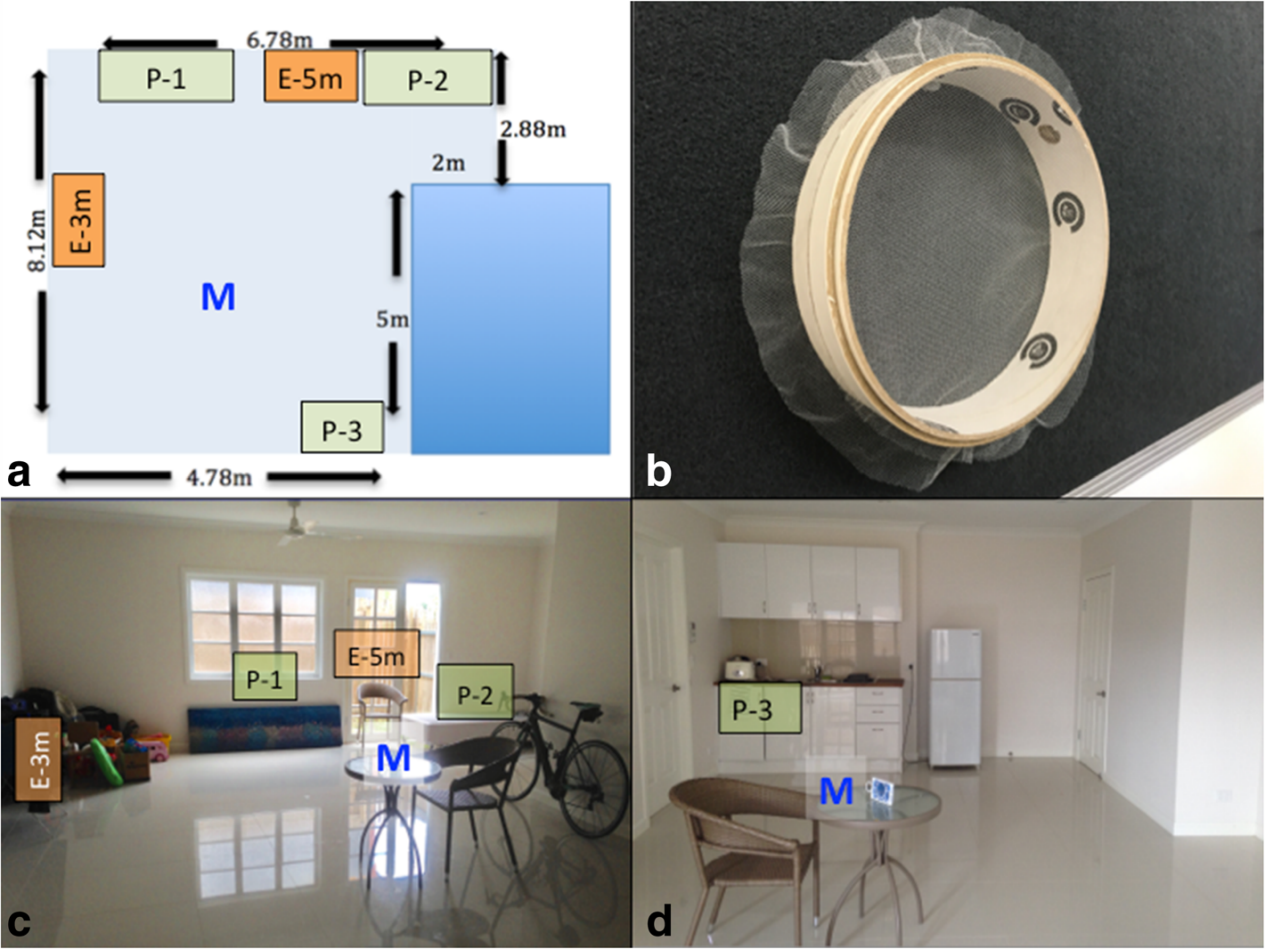
**a** Room set-up indicating *protected* and *exposed* locations within the room where tambourine cages of female *Ae. aegypti* were placed. “P” and “E” refer to *protected* and *exposed* locations, respectively. P-1 = behind large painting leaning against wall; P-2 = under the bed; and P-3 = inside the cupboard with door ajar; E-3 m = *exposed* 3 m from emanator; and E-5 m = *exposed* 5 m from the emanator. “M” indicates the location of the metofluthrin emanator. **b** Tambourine cage. **c-d** View of experimental set-up within the room indicating *protected* and *exposed* locations

Groups of five female *Ae. aegypti* were placed into separate tambourine cages (Fig. 5b) and placed in selected *exposed* and *protected* locations within a large room (Fig. 5a-d). *Exposed* locations were in plain sight and unsheltered, where *protected* locations were sheltered within the room (e.g. under a bed or behind an object leaning against a wall).

**Fig. 6:**
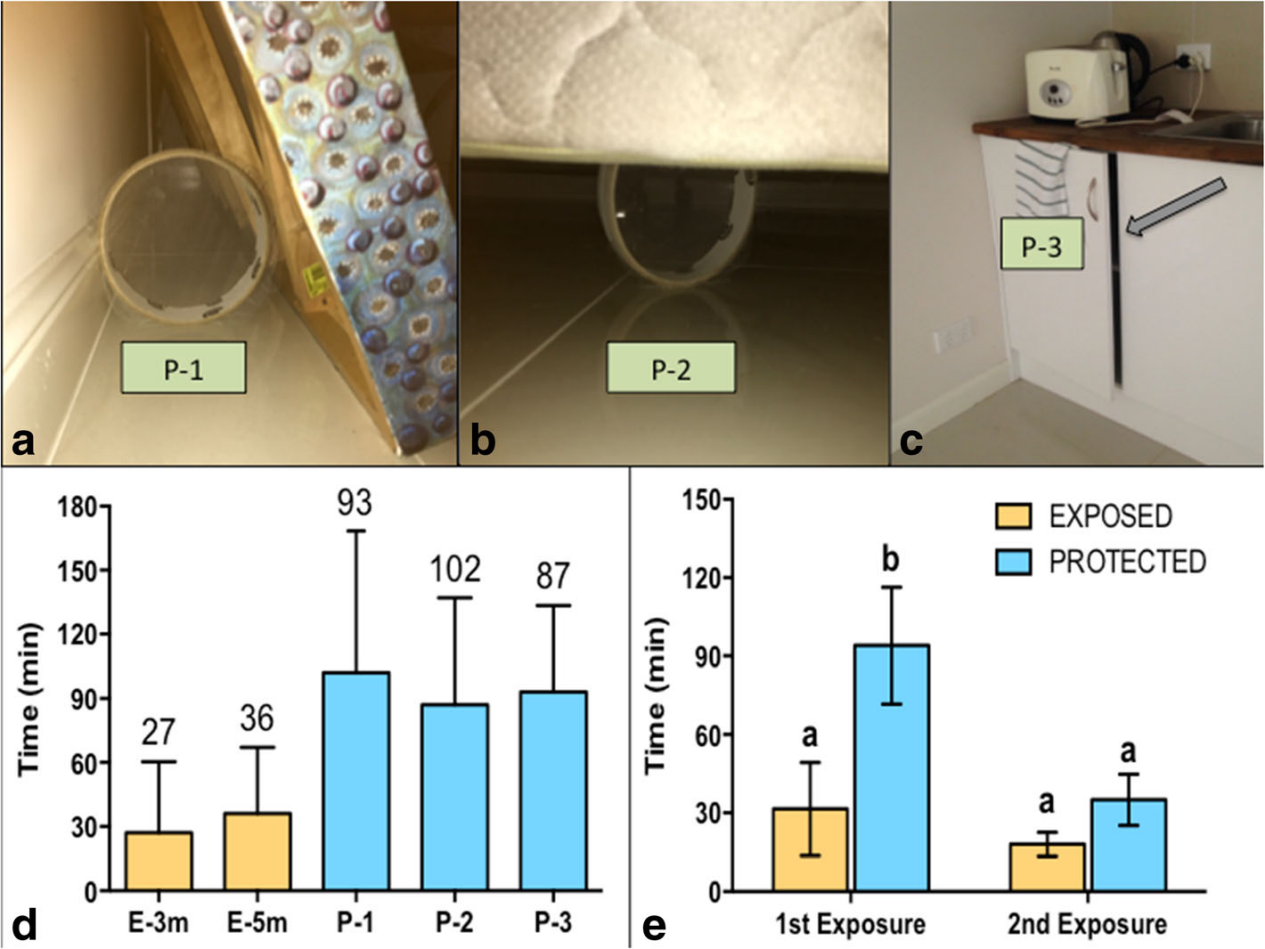
**a** The painting (P-1) leaning against wall with a tambourine cage behind it. **b** Tambourine cage location under the bed (P-2). **c** Cupboard ajar (P-3) containing tambourine cage. **d** Mean number of minutes (95% CIs) required for 100% KD to be achieved in each tambourine cage between locations in the first exposure. **E-3 m and E-5 m**: *exposed* cages at 3 and 5 m distance from the emanator, respectively; **P-1**: behind painting; **P-2**: under bed; **P-3**: in the cupboard with the door ajar. **e** Difference in the mean (95% CIs) number of minutes to 100% knockdown of mosquitoes between *exposed* and *protected* locations and the first and second exposure

Three *protected* locations were created within the room: (i) behind a large painting leaning against a wall (Fig. 6a); (ii) underneath a bed (Fig. 6b), and (iii) within a cupboard with its door 5 cm ajar (Fig. 6c). Two *exposed* locations were also included at 3 and 5 m distance from the emanator (Fig. 5a, c). Five female *Ae. aegypti* were kept in a tambourine cage in a separate untreated room as a control. Once in position, the metofluthrin emanator was activated in the room, and the time to 100% knockdown was recorded. One hundred percent knockdown was used as the indicator that the replication for the treatment (*protected* or *exposed*) was completed.

A ceiling fan on low setting (0.8 m/s) was used to aid in air circulation of the volatilizing compound. Using a flashlight, cages were visually assessed for knockdown every 15 min in order not to disrupt the tambourine cage or contaminate the airspace surrounding it. Once 100% of the mosquitoes were knocked down within a cage, it was immediately replaced with another cage of 5 mosquitoes, taking care not to disturb the *protected* space or expose the second cage prior to its placement. The second cage was observed every 15 min until 100% KD was achieved, and then removed. Between each replication, the bed and painting were moved and turned over, and the cupboard opened widely, to ensure the metofluthrin was evacuated from the *protected* spaces properly. Fans were turned on high and all doors and windows open for 2 h. A total of 5 replications were completed. The time to achieve 100% knockdown was compared between the *protected* and *exposed* locations for both the first and second exposure of tambourine cages. The second exposure allowed us to observe whether or not knockdown in the *protected* locations, over time, would be a result of a maintained low-dose exposure or if the concentration of the compound in the *protected* space would reach some level of equilibrium with the rest of the room. Five mosquitoes in a tambourine cage were kept in a separate room, outside of the treatment area as a control for each replicate. Data were analyzed using a negative binomial regression analysis in SPSS.

### Statistical analysis

Data were analyzed, depending on the model required for each analysis, in IBM® SPSS® version 24.0 or Prism 6 for Mac OSX (v. 6.0 h, GraphPad Software Inc.).

## Results

### Preliminary study: 22-h survival (fan off)

One hundred percent mortality was observed in *Ae. aegypti* exposed to a 10% AI metofluthrin emanator over a 22-h period in a largely treated room *versus* 2.7% mortality in the untreated room, indicating the high impact on mosquito mortality as a result of exposure to the metofluthrin emanator over a sustained period (*U* = 0, *P* = 0.0079),. The mean of the initial HLCs performed over a 5-min period prior to each control or treatment replication, were 81.4 and 70, respectively, confirming similar levels of activity and fitness of the mosquitoes used in both the treatment and control replications. (Fig. 2b, c).

### Effective spatial range of metofluthrin (fan off)

After a 10-min exposure period to the metofluthrin emanator, irrespective of the host’s distance from the metofluthrin emanator (1, 3 and 5 m), all mosquito host-seeking behaviour ceased in the presence of metofluthrin. A Mann-Whitney test found that the metofluthrin significantly impacted the host-seeking activity of the mosquitoes (*U* = 0, *P* < 0.0001). Host-seeking activity was observed during the control replicates, as indicated by the high values of HLCs taken over a 5-min period (Fig. 2e).

### The effect of host and metofluthrin location relative to harbourage areas on human landing counts (fan on vs fan off)

#### Fan off

A negative binomial regression analysis found that both the treatment (presence or absence of the metofluthrin) and the location of the host (*near* or *far* from the HA) were significant predictors of HLCs (*P* < 0.0001 and *P* = 0.006, respectively); however, an interaction between host location and treatment was not found. Biting activity, overall, was reduced by 78% in the presence of a 10% AI metofluthrin emanator, and the host was most likely to be bitten, regardless of the presence or absence of the metofluthrin, when located *near* the HA (within 1 m), opposed to being located *far* from the HA (8 m away), on the opposite end of the room (Fig. 3c). In the presence of an active emanator, host-seeking behaviour, although reduced, was detectable at 10 and 20 min post-activation with the fans off.

#### Fan on

When the same experiment (*Fan off*) was repeated with a ceiling fan on “low” setting (0.8 m/s airflow when standing directly below fan), mosquito host-seeking behaviour was reduced by 100% (*U* = 0, *P* < 0.0001) when a metofluthrin emanator was present in the room, regardless of its location in the room (Fig. 3d). In the absence of the metofluthrin emanator, the host was most likely to be bitten when located *near* the HA (within 1 m) (Fig. 3d).

### Introduction of a new mosquito to a treated room (fan off)

A Fisher’s exact test found that when mosquitoes were released into a treated room, they were less likely to find the host compared to mosquitoes released into an untreated room (*P* < 0.0001), $\overset{-}{X}=31.25\%$ and 100%, respectively (Fig. 4d). We also found that 100% of mosquitoes within the room reached the host in 76 s (*n* = 15), on average, and 26 s in a treated room (*n* = 5) (Fig. 4e). During the treatment, if the mosquito hadn’t found the host within the first 35 s of entering the room, it was unlikely to ever reach the host and attempt blood feeding.

### The impact of protected mosquito resting areas on efficacy (fan on)

Using a negative binomial regression analysis, a significant effect of *exposure type* was found on mosquito knockdown in *exposed versus protected* locations (*P* = 0.003). Overall, cages of mosquitoes in *exposed* locations of the room reached 100% knockdown at a significantly faster rate than those placed in *protected* locations, $\overset{-}{X}$= 23.96 min and $\overset{-}{X}$= 57.6 min, respectively (Fig. 6d). *Exposure round*, whether or not the cage of mosquitoes was the first or second cage at each location, was also a significant predictor in the time required to achieve 100% knockdown (*P* = 0.004), regardless of whether or not it was *exposed* or *protected*. The second cage placed at any location achieved 100% knockdown more rapidly than the first cage placed at the same location, $\overset{-}{X}$= 55.96 min and $\overset{-}{X}$= 24.66 min, respectively (Fig. 6e). A significant interaction was also observed between the two independent variables, *exposure type* and *exposure round* (*P* < 0.0001). Upon activation, the time required for metofluthrin to infiltrate *protected* resting locations within a room causing knockdown in caged mosquitoes, required more time than the *exposed* locations, $\overset{-}{X}=94\  \min\ \mathrm{and}\ \overset{-}{X}$= 31.5 min, respectively. In the second *exposure round*, the mean time required to achieve 100% knockdown in *protected* *versus* *exposed* locations was 35.5 mins and 18.8 mins, respectively, indicating an increase in the accumulation and infiltration of metofluthrin within the room and to the *protected* locations (Fig. 6e). The 5 control cages located in a separate untreated room maintained 0% KD throughout the duration of the experiment.

## Discussion

When an individual is particularly vulnerable during an arbovirus outbreak (e.g. pregnant women and ZIKV), they are dependent upon the effective implementation of vector control at all levels, including government and community. At the individual level, it is important that safe and effective products are accessible commercially as it allows the individual to take control over their own health, including their families.

From previous studies, we understand that metofluthrin has two main modes of action in reducing human biting: firstly, through confusion and disruption in orientation towards the host that stops biting; and secondly, through the knockdown and subsequent kill of the mosquito [21, 28, 31]. Here we explored the use of metofluthrin 10% AI in the household in order to better understand how to use this product within the home to protect the individual. It is important to note that the size of the rooms (111 m^3^ and 70 m^3^) that we used in these experiments were much larger than the rooms (approx. 25 m^3^) used in previous metofluthrin studies by Rapley et al. [21] and Ritchie & Devine [31].

The insecticidal properties of the metofluthrin on *Ae. aegypti* were confirmed here over a 22-h exposure period where we observed 100% mortality, similar to what was found by Rapley et al. [21] with a 4.1% AI emanator in a 25 m^3^ room. Without the assistance of a fan, when the metofluthrin emanator was centrally located within a large room, all biting ceased after 10 min of exposure within 5 m of the emanator. Earlier studies in a 25 m^3^ room found negligible biting after 8 min of exposure to a 10% AI metofluthrin emanator [31]. Given the size of our room, we had expected the mosquitoes to escape the effective range of the emanator, and for host-seeking activity to continue much longer than the 8 min observed by Ritchie et al. [31] in a 25 m^3^ room with a 10% AI formulation. A more recent study by Darbro et al. [32] using bioassay cages and hand landing counts in indoor spaces, found that irrespective of room size, after 10 min of exposure, landing rates were reduced by 50–90% and 25–90% at 1 m and 3 m, respectively. A result which also supports host-seeking activity in the presence of the metofluthrin emanator after 10 min. An extended observation in the same study found that in a 42 m^3^ room, probing behaviour continued at one meter from the emanator up to 6 h, and up to 53 h when the cage was set at three meters away. Based on the results of Darbro et al. one would assume that in our 111 m^3^ room, that some host-seeking activity would have persisted past 10 min, however, this was not the case. The results from Darbro et al. tend to underestimate the true efficacy of the 10% AI metofluthrin emanator with free-flying mosquitoes indoors. This could be explained by a decrease in airflow into bioassay cages, and therefore a subsequent delay in the effect of the metofluthrin on the mosquitoes.

When we discovered that the effective range of the metofluthrin was greater than we initially anticipated based on previous studies, we needed to increase the range of the emanator, Within the constraints of our large room, we were able to increase this range to eight meters. Here we used an HA to visually “bait” the mosquito to one end of the room while placing the metofluthrin emanator or host immediately next to or opposite it (eight meters), and always opposite each other. The HA encouraged harbouring of the mosquitoes immediately next to or opposite (eight meters distance) the emanator. With the fans off, host-seeking behaviour was reduced overall by 78% after 10 min of metofluthrin exposure, regardless of the location of the emanator or the host relative to the HA. A negligible level of host-seeking continued up to 20 min post-activation of the emanator. When we repeated the experiment with a ceiling fan (0.8 m/s), at 10 min post activation, all biting had ceased, illustrating the importance of air circulation when using metofluthrin in large spaces.

When we look at the primary gap that metofluthrin could fill when it comes to controlling vector-borne diseases transmitted by *Ae. aegypti*, it’s the protection of individuals from the bites of host-seeking *Ae. aegypti* that have newly entered a dwelling. For example, indoor residual spraying targets mosquito resting areas not flying mosquitoes that have newly entered a dwelling or room seeking a host. In this study, we found that previously unexposed mosquitoes released into a metofluthrin-treated room had a 31% frequency of reaching the host without seeking a resting site first, *versus* 100% success in reaching the host during the control. Of that 31.25%, we discovered that if the mosquito did not reach the host within 30 s of entering a treated room, they were unlikely ever to reach the host. The distance between the released mosquito and the host seated on the bed was approximately three meters within a completely white room with very few visual distractions, making it less challenging for the mosquito to find the host. While there is some risk of biting from quick entry, the room is essentially protected.

This brought us to ask the next question: is there anywhere within a treated room that a mosquito can remain *protected* from the effects of the metofluthrin, whereby at any time it could leave its small “pocket of protection” nearby a host, and attempt blood feeding? Within a household, there are objects, ranging from a bookshelf to a basket of laundry, and regardless of whether or not these items are organized or cluttered, they provide harbouring areas for *Ae. aegypti* in the home. We created three *protected* HAs that were common and where a mosquito could potentially reduce its exposure to metofluthrin in the home: behind an object leaning against a wall; under a bed; and within a cupboard that had its door slightly ajar. When we placed *Ae. aegypti* into these *protected* spaces and compared them to mosquitoes that were in *exposed* positions, we found that from the time of metofluthrin activation, there was a significant delay in effect between the *protected* mosquitoes and the *exposed,* indicating a potential lapse in protection. On average, the *exposed* and *protected* groups achieved 100% knockdown within 31 and 94 min, respectively, after activation of the metofluthrin. This suggests that mosquitoes in *protected* spaces took three times longer to receive the impacts of the metofluthrin from the time the emanator was activated. In the second exposure to the same emanator, the respective mean time to knockdown was shortened to 18 and 35 min for *exposed* and *protected* mosquitoes. This illustrates that there is a delay for these *protected* spaces to become infiltrated with metofluthrin, and in time the *exposed* and *protected* spaces will reach equilibrium, affecting mosquitoes equally throughout the room in terms of impacts on host-seeking and mortality.

The next stage of these metofluthrin studies is to investigate the efficacy of these devices on free-flying *Ae. aegypti* in the common areas around the house that are covered, but essentially outdoors (e.g. verandas) as well as rooms with open windows and airflow. An earlier study by Kawada et al. [33] in Vietnam found that a decrease in openings to a room treated with metofluthrin-impregnated plastic strips positively affected the spatial repellency of the metofluthrin. On a veranda setting in north Queensland, using a bioassay cage containing *Ae. aegypti*, a significant reduction in biting activity was observed at one meter from the metofluthrin emanator [32] and in outdoor field trials in the U.S., metofluthrin-impregnated paper emanators reduced biting in *Aedes vexans* between 95 and 97% when located at 1.2 m from the host [30]. These studies support and highlight the need for further field trials on the effects of metofluthrin on *Ae. aegypti* in semi-outdoor areas as well as indoor rooms with open doors and windows.

## Conclusions

Metofluthrin is very effective in interrupting the host-seeking abilities of female *Ae. aegypti* within a short period of time and over an extensive area indoors. In future outbreaks of viruses transmitted by *Ae. aegypti*, metofluthrin may play an important role in personal protection and disease control, particularly in instances where large-scale operations are exhausted, and vulnerable individuals need to be targeted.

Within the limits of this study, we have been able to describe the strengths and limitations of this novel compound. From this, several recommendations for use are apparent. First, without fans, metofluthrin works very well, however, in these instances, it is important that the emanator remains centrally located within a room. Secondly, to increase efficacy, particularly when looking at covering larger spaces, metofluthrin is most efficiently distributed with the aid of a fan. Thirdly, when newly treating a room with metofluthrin, it is best to allow a period of 2–4 h of exposure to ensure a sufficient distribution of the compound throughout the room and to its protected or sheltered areas within it where mosquitoes may be resting.

## Abbreviations

CHKV: chikungunya virus
DENV: dengue fever virus
HA: harbourage area
IRS: indoor residual spraying
SE: standard error
YFV: yellow fever virus
ZIKV: Zika Virus
